# Germline sequence variants contributing to cancer susceptibility in South African breast cancer patients of African ancestry

**DOI:** 10.1038/s41598-022-04791-1

**Published:** 2022-01-17

**Authors:** Dewald Eygelaar, Elizabeth J. van Rensburg, Fourie Joubert

**Affiliations:** 1 Department of Biochemistry, Genetics and Microbiology, University of Pretoria, Pretoria, 0001 South Africa; 2 Centre for Bioinformatics and Computational Biology, University of Pretoria, Pretoria, 0001 South Africa; 3 Genomics Research Institute, University of Pretoria, Pretoria, 0001 South Africa; 4 Genetics Division, University of Pretoria, Pretoria, 0001 South Africa

**Keywords:** Cancer genetics, Breast cancer

## Abstract

Since the discovery of the breast cancer susceptibility genes, *BRCA1* and *BRCA2,* various other genes conferring an increased risk for breast cancer have been identified. Studies to evaluate sequence variants in cancer predisposition genes among women of African ancestry are limited and mostly focused on *BRCA1* and *BRCA2*. To characterize germline sequence variants in cancer susceptibility genes, we analysed a cohort of 165 South African women of self-identified African ancestry diagnosed with breast cancer, who were unselected for family history of cancer. With the exception of four cases, all others were previously investigated for *BRCA1* and *BRCA2* deleterious variants, and were negative for pathogenic variants. We utilized the Illumina TruSight cancer panel for targeted sequencing of 94 cancer susceptibility genes. A total of 3.6% of patients carried a pathogenic/likely pathogenic variant in a known breast cancer susceptibility gene: 1.2% in *BRCA1*, 0.6% in each of *BRCA2*, *ATM, CHEK2* and *PALB,* none of whom had any family history of breast cancer. The mean age of patients who carried deleterious variant in *BRCA1/BRCA2* was 39 years and 8 months compared to 47 years and 3 months among women who carried a deleterious variant in other breast cancer susceptibility genes.

## Introduction

Breast cancer is an increasing public health problem worldwide. It is the most commonly diagnosed cancer and the leading cause of cancer deaths in women. Breast cancer incidence and mortality rates are rising in transitioning countries in Africa, with some of the most rapid increases occurring in sub-Saharan Africa^1,2^. The GLOBOCAN 2020 database of the International Agency for Research on Cancer (IARC), estimated the current age standardised breast cancer incidence per 100,000 women in Southern (50.4), Western (41.5), Eastern (33), and Central Africa (32.7) with associated mortality rates estimated at 15.7, 22.3, 17.9, and 18, respectively^3^. Female breast cancer represents 25.8% of all cancer diagnoses in sub-Saharan Africa^1,2^. Newly diagnosed breast cancer cases in South Africa accounts for 27.1% of female cancers in 2020, with age-standardized (World) incidence and mortality rates of 52.6 and 16 (per 100,000 women) respectively^3^. Cancer results from a process of genetic changes, some inherited, some induced by environmental exposures and some occurring by chance. Early age of onset and a family history is a hallmark of hereditary breast cancer that is associated with germline variants in the high-penetrance genes, *BRCA1* and *BRCA2*^4–6^. An association with breast cancer susceptibility has also been reported for a further eleven high- to moderate-penetrance genes (*TP53, PALB2, PTEN, STK11, CDH1, ATM, BRIP1, CHEK2, RAD51B, RAD51C,* and *RAD51D*)^7,8^. In addition, pathogenic variants in genes from the mismatch repair pathway (*MLH1, MSH2, MSH6* and *PMS2*) have been identified in breast cancer and ovarian cancer patients^7^. With the advent of next-generation sequencing (NGS), simultaneous sequencing of multiple cancer susceptibility genes is available through multiplex panels. Several studies utilizing NGS gene panels have been carried out, mainly on breast cancer cases from west European and Asian populations^9,10^. Some studies have included African-Americans, but this data can be difficult to interpret in an African context due to the fact that they are an admixed population. The estimated proportion of African, European and Native American ancestry in African-American groups vary from 76 to 85% African, 14% to 21% European and 1% to 3% Native American ancestry^11^.

Studies of breast cancer susceptibility genes in African populations are scarce and have mainly focused on *BRCA1* and *BRCA2*^12^. In addition, there is a paucity of data on sequence variants in cancer predisposition genes among women of African descent/ancestry with breast cancer, who are unselected for age at diagnosis, or family history of cancer. To date only two studies in Africa, one on Nigerian women^13^, and one on women from Uganda and Cameroon^14^, have used multigene panel sequencing to test for germline variants in patients, unselected for family history or age at diagnosis. In the present study, we included South African women of African ancestry (self-identified) diagnosed with breast cancer, who were unselected for age at diagnosis or family history of cancer. With the exception of four cases, all others were previously investigated for *BRCA1* and *BRCA2* pathogenic variants, and were negative for pathogenic/likely pathogenic *BRCA1/BRCA2* variants We used targeted next-generation sequencing of a multigene panel, comprised of 94 cancer susceptibility genes (Illumina TruSight cancer panel) in order to assess the frequency of deleterious germline variants in this cohort.

## Results

**Figure 1:**
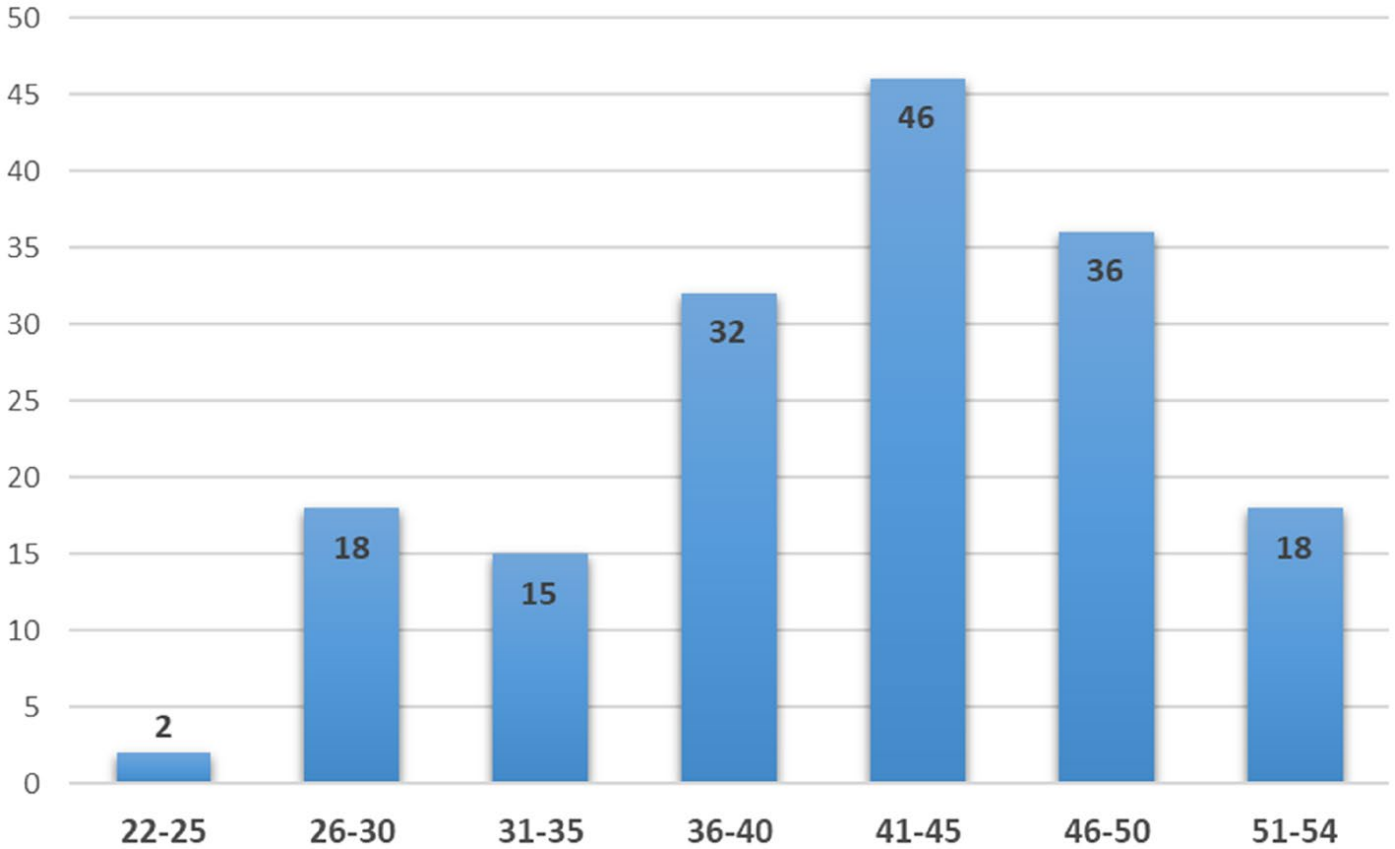
Distribution of patient age at first breast cancer diagnosis displayed in 5 year intervals (Figure generated using Microsoft Excel).

A total of 165 breast cancer patients of African ancestry (self-reported), unselected for family history or age at diagnosis, were included in this study (Supplementary Table S1). Their mean age (SD) at diagnosis was 41.28 (7.35) years (age range 22 to 54 years). Figure 1 depicts the patients’ age at diagnosis displayed in 5-year intervals. Furthermore, 9% (15/165) of the patients reported either a 1st and/or 2nd degree relative with breast and/or ovarian cancer (Supplementary Table S1).

Information on the histology type was available for 145 of the 165 patients. The most common type was infiltrating ductal carcinoma (81.8%), followed by medullary ductal carcinoma (2.4%), invasive lobular carcinoma (1.2%), and at 0.6% each, tubular ductal carcinoma, papillary carcinoma, infiltrating mucinous carcinoma and carcinoma not otherwise specified. Cancer grade information was unavailable for eight of the 165 patients. Only one of the patients was diagnosed with grade I (0.6%), 40 (24.2%) with grade II, 46 (27.9%) with grade III and 70 (42.4%) with grade IV breast cancer. High-grade tumours (grade III and IV) were by far the most common, accounting for 70.3% of all carcinomas. Information from hospital and histology records regarding hormone receptor status was unavailable.

We performed targeted sequencing of 94 cancer susceptibility genes (Supplementary Table S2) in peripheral blood DNA samples from the 165 patients, using a next-generation sequencing platform. All of the 94 genes were investigated for nonsense, frameshift, or splice-site variants affecting the invariant splice sites. In addition, a subset of nineteen established and candidate breast or ovarian cancer genes (*ATM, BARD1*, *BRCA1*, *BRCA2*, *BRIP1, CDH1*, *CHEK2, MLH1, MSH2, MSH6, NBN*, *NF1, PALB2*, *PMS2, PTEN*, *RAD51C*, *RAD51D*, *STK11* and *TP53*) were investigated for all sequence variants.

### Variants

**Figure 2:**
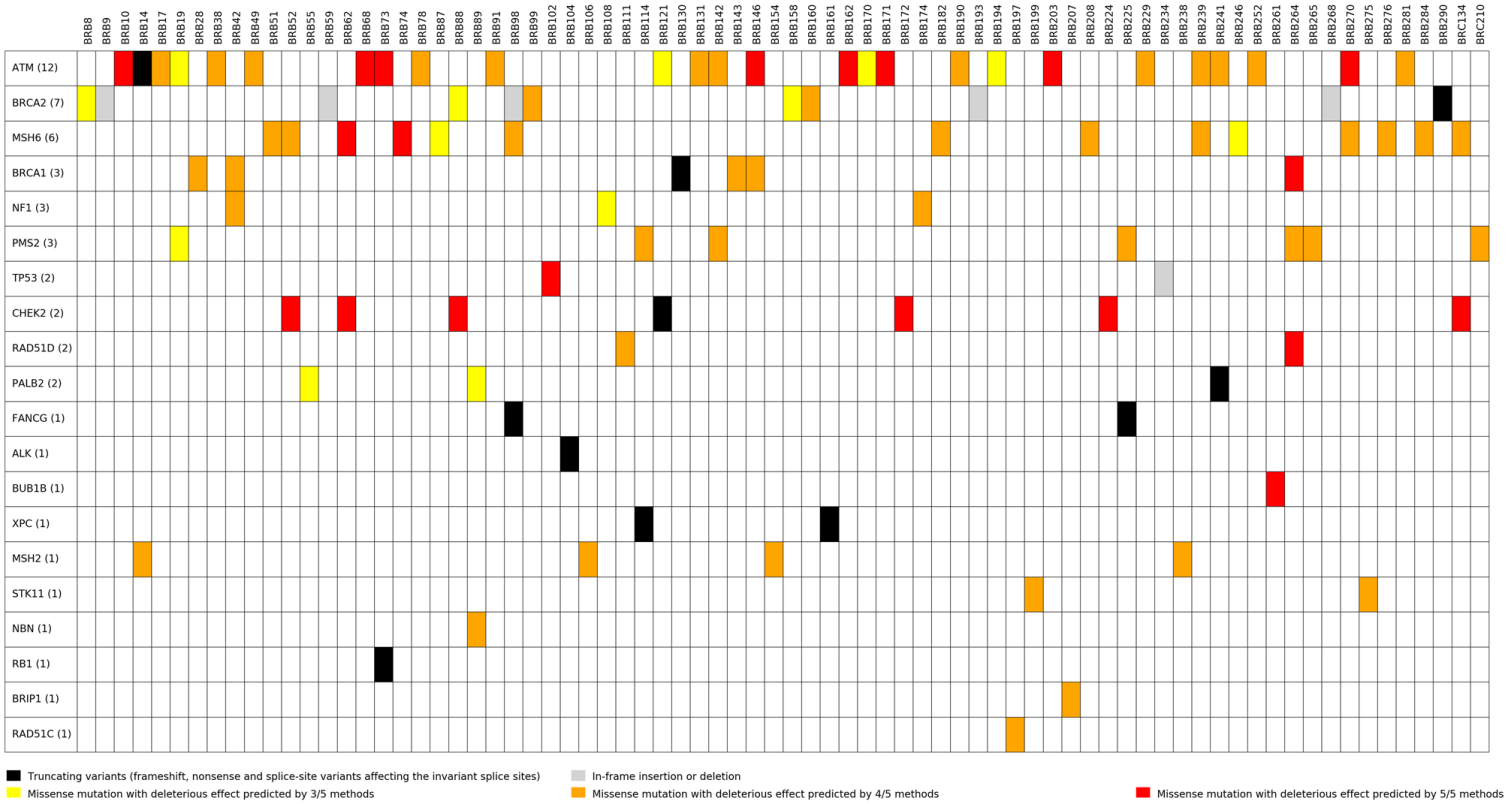
Matrix of patients vs. genes with sequence variants in breast cancer susceptibility genes and genes exclusively investigated for truncating variants (multiple variants per gene may be present). The genes are sorted from the most to least number of variants per gene as indicated in brackets. Black indicates truncating variants (frameshift, nonsense and splice-site variants affecting the invariant splice sites); Grey indicates an in-frame insertion or deletion. Missense variants are indicated according to the five in silico functional effect predictors, where yellow indicates a deleterious effect predicted by 3/5 methods, orange 4/5 methods and red 5/5 methods (Figure generated using Matplotlib 3.4.2: https://matplotlib.org).

In the final data set, filtering by concordant deleterious effect prediction for missense variants in the breast cancer susceptibility genes (at least 3/5 methods), population allele frequencies (< 1% in African populations of 1000 genomes phase 1 and 3), read depth (≥ 20), resulted in the identification of 52 unique variants in 20 genes. Of these 52 variants, eleven were classified as PV/LPV (Table 1), 27 classified as VUSs (Table 2) and 14 were classified as benign/likely benign (Supplementary Table S3). These variants were present in 76 of the patients (Fig. 2, Supplementary Table S4). Missense variants dominated (39), followed by frameshift variants (3), nonsense variants (3), variants affecting canonical splice sites (3), and in-frame deletions (3).

**Table 1:** Pathogenic/likely pathogenic variants detected in a South African breast cancer cohort of African ancestry.

Gene (RefSeq)*	Nucleotide change	Location	Predicted protein consequence	dbSNP	Patient	Age at diagnosis (years:months)
**Pathogenic/ likely pathogenic variants in known breast cancer susceptibility genes**						
*ATM (NM_000051.3)*	c.162 T > A^**#**^	exon 3	p.Tyr54Ter	–	BRB14	47:8
*BRCA1 (NM_007294.3)*	c.4524G > A	exon 15	p.Trp1508Ter	rs80356885	BRB130	45:8
	c.5096G > A	exon 18	p.Arg1699Gln	rs41293459	BRB264	42:3
*BRCA2 (NM_000059.3)*	c.5771_5774del	exon 11	p.Ile1924ArgfsTer38	rs80359535	BRB290	26:6
*CHEK2 (NM_001005735.1)*	c.283C > T	exon 2	p.Arg95Ter	rs587781269	BRB121	54:0
*PALB2 (NM_024675.3)*	c.2835-1G > C	intron 8	p.(?)	rs515726099	BRB241	40:1
**Pathogenic variants in hereditary cancer predisposition genes exclusively investigated for truncating variants**						
*ALK (NM_004304.4)*	c.2782dup^**#**^	exon 16	p.Cys928LeufsTer20	–	BRB104	47:0
*BUB1B (NM_001211.5)*	c.2848C > T^**#**^	exon 1	p.Gln950Ter	–	BRB261	38:1
*FANCG (NM_004629.1)*	c.637_643del	exon 5	p.Tyr213LysfsTer6	rs587776640	BRB225	34:4
					BRB98	43:3
*RB1 (NM_000321.2)*	c.1127 + 1G > A^**#**^	intron	p.(?)	–	BRB73	29:11
*XPC (NM_004628.4)*	c.2251-1G > C	intron 13	p.(?)	rs754673606	BRB114	47:1
					BRB161	29:6

*Reference sequences obtained from the NCBI database. For *BRCA1* the most common human transcript (NM_007294.3) was used with custom numbering of the exons (missing exon 4). Variant nomenclature according to the Human Genome Variation Society (HGVS) where complimentary DNA (cDNA) numbering + 1 corresponds to the A of the ATG translation initiation codon.

^#^Not reported in dbSNP (http://www.ncbi.nlm.nih.gov/SNP), EVS (http://evs.gs.washington.edu/EVS), gnomAD (https://gnomad.broadinstitute.org) or ClinVar (https://www.ncbi.nlm.nih.gov/clinvar).

**Table 2:** Variants of unknown clinical significance identified in established and candidate breast or ovarian cancer genes in a South African Breast cancer cohort of African ancestry.

Gene (RefSeq)*	Variant	Exon	Predicted protein change	dbSNP	Patient	Age (years:months)
*ATM* (NM_000051.3)	c.131A > G	Exon 3	p.Asp44Gly	rs150143957	BRB146	52:2
					BRB38	46:10
					BRB49	43:4
*ATM*	c.320G > A	Exon 4	p.Cys107Tyr	rs142358238	BRB171	37:4
					BRB68	43:0
*ATM*	c.1358C > T	Exon 10	p.Pro453Leu	rs786204124	BRB121	54:0
					BRB170	40:2
					BRB194	44:1
*ATM*	c.3078G > C^#^	Exon 21	p.Trp1026Cys	–	BRB146	52:2
*ATM*	c.4329C > A	Exon 29	p.His1443Gln	rs377065665	BRB131	45:3
					BRB17	44:11
					BRB229	38:11
					BRB281	51:0
					BRB78	36:4
*ATM*	c.6176C > T	Exon 42	p.Thr2059Ile	rs144761622	BRB239	51:11
					BRB241	40:1
					BRB252	40:9
*ATM*	c.6194T > C	Exon 42	p.Ile2065Thr	rs372838622	BRB19	36:0
*ATM*	c.8558C > T	Exon 58	p.Thr2853Met	rs141534716	BRB10	42:8
					BRB162	42:10
					BRB203	39:7
					BRB270	41:2
					BRB73	29:11
*BRCA2* (NM_000059.3)	c.4798_4800del	Exon 11	p.Asn1600del	rs276174851	BRB193	43:5
					BRB268	46:2
					BRB98	43:3
*BRCA2*	c.7762A > G^#^	Exon 16	p.Ile2588Val	–	BRB158	53:7
*BRCA2*	c.8390A > G^#^	Exon 19	p.Asp2797Gly	–	BRB8	28:0
*BRCA2*	c.9088A > C^#^	Exon 23	p.Thr3030Pro	–	BRB88	39:3
*BRIP1* (NM_032043.2)	c.2131A > G	Exon 15	p.Thr711Ala	rs760515227	BRB207	49:9
*MSH2* (NM_000251.2)	c.508C > G	Exon 3	p.Gln170Glu	rs63750843	BRB106	35:0
					BRB14	47:8
					BRB154	51:2
					BRB238	44:10
*MSH6* (NM_000179.2)	c.560A > G^#^	Exon 3	p.Lys187Arg	–	BRB246	42:1
*MSH6*	c.2083C > T^#^	Exon 4	p.Leu695Phe	–	BRB182	41:2
					BRB208	44:8
					BRB284	30:10
					BRB98	43:3
*MSH6*	c.2347 T > A	Exon 4	p.Cys783Ser	rs373721483	BRB74	47:4
*MSH6*	c.2962C > T	Exon 4	p.Arg988Cys	rs61753795	BRB62	43:7
*MSH6*	c.3489A > C	exon 6	p.Glu1163Asp	rs531674673	BRB239	51:11
					BRB270	41:2
					BRB276	46:6
					BRB51	52:6
					BRB52	46:11
					BRC134	45:11
*NBN* (NM_002485.4)	c.706A > G	Exon 7	p.Lys236Glu	rs1060503482	BRB89	48:8
*NF1* (NM_001042492.2)	c.4943C > T	Exon 37	p.Thr1648Ile	rs376655102	BRB174	37:8
					BRB42	49:11
*PALB2* (NM_024675.3)	c.23C > T	Exon 1	p.Pro8Leu	rs150390726	BRB55	32:9
					BRB89	48:8
*RAD51C* (NM_058216.2)	c.779G > A	Exon 5	p.Arg260Gln	rs730881926	BRB197	51:0
*RAD51D* (NM_002878.3)	c.250A > G	Exon 3	p.Thr84Ala	rs200018296	BRB111	42:7
*STK11* (NM_000455.4)	c.888G > C	Exon 7	p.Lys296Asn	rs1555738868	BRB199	45:2
					BRB275	47:4
*TP53* (NM_000546.5)	c.476C > T	Exon 5	p.Ala159Val	rs1555526131	BRB102	40:9
*TP53*	c.393_395del	Exon 5	p.Asn131del	rs879254214	BRB234	39:10

Variants are named according to the Human Genome Variation Society (HGVS) nomenclature, where complimentary DNA (cDNA) numbering + 1 corresponds to the A of the ATG translation initiation codon.

*Reference sequences obtained from the NCBI database. For *BRCA1* the most common human transcript (NM_007294.3) is used with custom numbering of the exons (missing exon 4).

^**#**^Not reported in dbSNP (http://www.ncbi.nlm.nih.gov/SNP), EVS (http://evs.gs.washington.edu/EVS), gnomAD (https://gnomad.broadinstitute.org) or ClinVar (https://www.ncbi.nlm.nih.gov/clinvar).

The concordance of the five in silico functional effect predictors is shown in Supplementary Fig. S1 (full concordance for 22 variants), and the number of distinct deleterious variants per predictor is shown in Supplementary Fig. S2.

#### Deleterious variants

Six patients (3.6%) were found to carry a pathogenic or likely pathogenic (P/LP) variant in one of five known breast cancer susceptibility genes: 1.2% in *BRCA1*, 0.6% in each of *BRCA2*, *ATM, CHEK2* and *PALB.* A further seven patients carried deleterious variants in one of five hereditary cancer predisposition genes exclusively investigated for truncating variants, specifically *ALK, BUB1B*, *FANCG*, *RB1* and *XPC* (Table 1). None of these patients reported any family history of cancer.

#### Variants of uncertain clinical significance

A total of 27 variants were detected in 12 genes associated with breast cancer susceptibility (Table 2). VUS were identified most commonly in the *ATM* gene (eight variants), followed by *MSH6* with five variants and *BRCA2* (four variants).

## Discussion

This study screened 165 South African breast cancer patients of African ancestry (self-identified) for the presence of deleterious germ line sequence variants in 94 genes associated with hereditary cancer. The patients were unselected for age at diagnosis or family history of cancer. With the exception of four cases (BRB130, BRB290, BRC134 and BRC210) all others were previously screened for *BRCA1/BRCA2* variants, and found to be negative for pathogenic/likely pathogenic variants.

Although the patients were unselected for family history of breast or ovarian cancer, 9% did report some family history of breast or ovarian cancer. This is higher than that reported for similar studies in breast cancer patients from Cameroon/Uganda (6.6%) and Nigeria (6%)^13,14^. With regards to tumour stage, 70.3% of patients were diagnosed with stage III/IV at diagnosis. It is thought that low survival rates in sub-Saharan Africa is mostly attributable to late-stage presentation. The stage at presentation of our cohort is similar to that reported in 83 studies across 17 sub_Saharan African countries, with 77% of cases presenting at stage III/IV^15^.

We identified pathogenic/likely pathogenic variants (P/LP) in 13 patients, in ten different genes (Table1), which represents 7.9% of the cohort. Six of these patients (3.6%) have P/LPVs in genes that are confirmed to confer an increased risk for breast cancer. The mean age of patients who carried deleterious variant in *BRCA1/BRCA2* was 39 years and 8 months compared to 47 years and 3 months among women who carried a deleterious variants in other breast cancer susceptibility genes. Pathogenic variants in non-*BRCA1/BRCA2* breast cancer susceptibility genes accounted for 1.8% of our cohort. None of these women reported any family history of cancer. In addition, 14 benign/likely benign variants were detected in eight breast cancer genes (Supplementary Table S3) and 27 VUS including six variants not previously described, were detected in 12 established and candidate breast cancer genes (Table 2).

In the studied cohort, variants in the *ATM* gene were the most frequently identified (Tables 1, 2). Pathogenic *ATM* variants act in a recessive manner to cause Ataxia telangiectasia (a neurodegenerative disease), whereas heterozygous carriers are at moderately increased risk for breast cancer^16,17^. Patient BRB14 (Zulu-speaking patient), diagnosed with breast cancer at age 47 years and 8 months, was a carrier of the novel *ATM* likely pathogenic variant, c.162T > A. It is predicted to be a nonsense variant, p.(Tyr54Ter), that may cause the transcript to be exposed to nonsense-mediated mRNA decay. If ATM is synthesized it will lack most of the protein sequence and thus be non-functional. Interestingly, a recent study that explored the clinico-pathologic characteristics of breast cancers developed by *ATM* mutation carriers reported the median age at first diagnosis to be 46.9 years in their cohort^18^. Unfortunately we do not have any further histopathologic information on the breast cancer of BRB14.

There has been some debate on whether mono-allelic truncating *ATM* variants are associated with increased breast cancer risk. Early on it was hypothesised that some missense variants in *ATM* might have dominant negative effects and confer a particularly high risk of breast cancer when heterozygous, compared to truncating variants^19^. In a meta-analysis of *ATM* variants, a later study found strong evidence that a subset of rare evolutionary unlikely missense variants confer increased cancer risk. They found marginal evidence that protein-truncating and splice-site variants contribute to breast cancer risk^20^. Goldgar et al*.*^21^ further investigated the issue and reported risk estimates that women who carry either a pathogenic missense or truncating variant have a significantly increased risk of breast cancer. To obtain accurate risk estimates require a large sample size, which a recent large study of more than 113,000 women (mostly population-based samples), addressed^22^. This study identified *ATM* protein-truncating variants to confer significant disease risks (odds ratio 2.1), compared to rare missense variants (odds ratio 1.06)^22^.

Two of the four patients (BRB130 and BRB290) who had not previously been screened for *BRCA1/BRCA2* variants, were found to carry a *BRCA1* or *BRCA2* deleterious variant (Table 1). The *BRCA1* c.4524G > A p.(Trp1508Ter) variant was identified in BRB130, a Tswana-speaking woman diagnosed with breast cancer at age 45 years and 8 months. The variant is predicted to introduce a stop codon that will produce a transcript that may be targeted for nonsense-mediated mRNA decay (NMD). This nonsense variant has been detected in multiple families with hereditary breast ovarian cancers^23–30^. Of note, the variant is also designated as 4643G > A in published literature.

BRB264 (diagnosed at 42 years and three months, Tsonga-speaking patient) carried the *BRCA1* c.5096G > A p.(Arg1699Gln), intermediate risk variant. It is located in the BRCA1 carboxyl terminal region of the transcriptional transactivation domain. The cancer risks associated with this variant was first defined by the ENIGMA consortium (Evidence-based Network for the Interpretation of Germline Mutant Alleles) in 2012 and in a follow up study in 2017 the risk estimates were confirmed^31,32^. Functional assays showed this variant to have impaired homology-directed DNA repair activity and it was classified as being a hypomorphic allele^33^. Interestingly, this pathogenic missense was also found in a Nigerian woman with breast cancer^13^.

The *BRCA2* frameshift variant, c.5771_5774del p.(Ile1924ArgfsTer38), was identified in BRB290 who was diagnosed with breast cancer at 26 years and 6 months of age. The variant is expected to result in loss of function due to an absent or disrupted protein. This alteration has been reported in multiple individuals (of European ancestry) with hereditary breast and ovarian cancer syndrome^34^ and has been reported as a founder mutation in Bantu-speaking Xhosa women from the Western Cape of South Africa^35^. BRB290 is however a Bantu-speaking Sotho individual, and at this time it is not possible to do any haplotype analysis to ascertain whether she carries this PV on the same haplotype as that of the Xhosa founder variant.

The pathogenic *CHEK2,* c.283C > T p.(Arg95Ter), variant detected in BRB121 (diagnosed at 54 years, Zulu-speaking patient) was previously identified in the germline of two Norwegian patients diagnosed with locally advanced breast cancer^36^. Of interest, both patients were resistant to anthracycline therapy. In vitro assays of the p.(Arg95Ter) variant found the CHEK2 protein to be non-functional in terms of kinase activity and dimerization. Loss of heterogeneity (LOH) analysis of the tumours found that the wild type allele of the *CHEK2* gene was lost for both of the patients^36^. The possibility that this nonsense variant together with LOH is associated with resistance to anthracyclines in cancer patients underlines its potential clinical importance. In a follow up case control study of 7081 incident cancer cases from Norway, Knappskog et al., detected the p.(Arg95Ter) variant in 0.23% breast cancer cases and in 0.16% prostate cancer cases^37^. This variant is also reported as pathogenic by multiple laboratories in ClinVar (Variation ID: 140772). In our study 0.61% (1/165) of cases carried a pathogenic *CHEK2* variant. There is substantial variation in the prevalence of germline *CHEK2* pathogenic variants among different populations and ethnicities, with individuals of European ancestry that have the highest prevalence^38^. A multi-ethnic population-based study of a cohort of breast cancer and ovarian cancer patients found that for breast cancer 2.3% (95% CI 1.8% to 2.8%) of white individuals and only 0.15% (95% CI 0% to 0.82%) of black individuals carried a pathogenic *CHEK2* variant^39^.

The *PALB2* variant, c.2835-1G > C, located in a canonical acceptor splice-site (in Intron 8) was identified in a Xhosa-speaking patient (BRB241, diagnosed at 40 years of age). The variant has been reported in the literature in persons affected with breast or ovarian cancer^40–43^. Several in silico bioinformatic tools predicted this variant to abolish the 3′-acceptor splice site, which would alter the natural splicing of *PALB2*. The expected effect is an in-frame deletion in the *PALB2* mRNA by skipping exon nine (deletion of 162 bp, 54 amino acids; Ala946 to Gly999). Another possibility is that an alternative cryptic splice site could be used. The strongest alternative site is in exon nine at c.2864, and should this be used, the result would be the loss of 30 bp (10 amino acids; Ala946 to Glu955) from exon nine.

cBROCA analysis of mRNA from patients with the c.2835-1G > C variant showed that it preferentially leads to skipping of exon 9 (r.2835–2996) and is therefore expected to produce an abnormal PALB2 protein, lacking the 54 amino acids^44^. The deleted section is part of the second and third blades of the WD40 domain of PALB2. This seven bladed region is essential for the interaction of BRCA2 with PALB2^45,46^. When BRCA2 is unable to bind to PALB2, homologous recombination repair is severely disrupted.

Pathogenic variants in five “other” cancer predisposition genes (*ALK, BUB1B, FANCG, RB1* and *XPC*) exclusively investigated for truncating variants, were identified in seven patients (Table 1). Three of the genes (*BUB1B, FANCG* and *XPC*) are associated with autosomal recessive conditions, requiring the inheritance of two pathogenic variants for the particular condition to manifest. Deleterious variants, either in the hetero- or homozygous state, in these genes have not been found to confer an increased risk for breast cancer. Pathogenic germline variants of *ALK* usually are gain-of-function missense variants that are associated with familial neuroblastoma^47,48^—the novel variant found in our study leads to a loss-of-function effect. Biallelic pathogenic variants in the spindle assembly checkpoint gene, *BUB1B,* causes the disorder, mosaic variegated aneuploidy (MVA)^49^. The *FANCG* frameshift variant, c.637_643del p.(Tyr213LysfsTer6), detected in two breast cancer patients (BRB98 & BRB225), is a founder variant that is present in 82% of Fanconi anaemia subtype G patients from sub-Saharan African populations^50^. The *RB1* gene is the first tumour suppressor gene to be cloned and germline pathogenic variants predispose to hereditary retinoblastomas (childhood retinal cancer)^51^. It is unknown whether BRB73, carrier of the *RB1* donor splice site variant, had retinoblastoma as a child. The *XPC* splice acceptor site variant, c.2251-1G > C, that was detected in two breast cancer patients (BRB114 & BRB161), is an ancient founder variant that is thought to have occurred ~ 800 years ago in the Bantu population of West-Central Africa^52^. This variant is present in the homozygous state in many Xeroderma pigmentosum families of African ancestry^52^.

Two of the VUS detected in the *ATM* gene (p.Asp44Gly, p.Glu2181Asp) were also found in breast cancer patients from Cameroon and Uganda^14^. The available evidence is currently insufficient to unequivocally determine the role of the VUS that we detected in established and candidate breast cancer genes (Table 2). However there is one variant of note, the PALB2 N-terminus variant c.23C > T p.(P8L) detected in two patients (BRB55 & BRB89). This variant is near the coiled-coil domain of PALB2 that is involved in hetero-dimerization of BRCA1 with the protein. PALB2 is an essential component in homologous recombination-based DNA repair (HR) and loss of PALB2 function was shown to be synthetic lethal in combination with poly(ADP-ribose) polymerase inhibitors (PARPi)^53,54^. This has led to the development of tests that exploit this weakness to assess the functional effect of *PALB2* sequence variants.

Functional assays that test the vulnerability of PALB2 variants to PARP inhibitors as well as HR functionality were applied to the p.(P8L) variant. Moderate but statistically significant (P < 0.0001) PARPi sensitivity was observed (76% cell survival), whereas wild type PALB2 had 100% cell survival^55^. The homology-directed repair assay found p.(P8L) to have an intermediate phenotype with a 40% reduction in HR when compared to wild type PALB2^55,56^, all of which appear to indicate that this variant may play a role in breast cancer.

A limitation of this study is that no copy number variation using NGS data or MLPA was used to investigate the genes. Thus large deletions or duplications could be undetected. Furthermore, the relatively small sample size and unavailability of hormone receptor status precluded any investigation of the prevalence of sequence variants by breast cancer subtype.

While precision medicine is currently still mostly out of reach in African countries due to economic reasons, the rapidly declining costs of genomic technologies will in future necessitate population-specific variant information, particularly in diseases such as cancer.

To our knowledge, this is the first study that has investigated South African breast cancer patients of African ancestry for germline sequence variants in a multigene panel.

Although we investigated a relatively small cohort of patients, our study provides some insights towards the genetic breast cancer risk factors in South African women of African ancestry. In conclusion, our study has shown that the 3.6% of women who carry a pathogenic/likely pathogenic variant in a breast cancer susceptibility gene do not necessarily have a family history of breast cancer. In our cohort there was an equal proportion of women who carried a deleterious variant in *BRCA1/BRCA2* (1.8%) and women who carried a deleterious variant in other breast cancer susceptibility genes (1.8%). These findings must however be treated with caution because of the small sample size. Further studies of a larger patient cohort is warranted to assess the distribution of variants in clinically relevant cancer susceptibility genes.

## Patients and methods

### Patients and DNA samples

Peripheral blood samples were previously collected from South African women with breast cancer, who attended the Oncology Clinic at Steve Biko Hospital, Pretoria, between 1993 and 2001. The study population were of self-reported African ancestry, at least 18 years old and were included regardless of age at diagnosis or family history. In total we received blood samples from 286 patients with age at diagnosis ranging from 21 to 85 years (mean 49.52 years ± 12.93 years). DNA was extracted from the blood samples using the method described by Johns and Paulus-Thomas^57^. For the current study we selected 165 of these patients (Supplementary Table S1) beginning with the youngest patients. With the exception of four cases (BRB130, BRB290, BRC134 and BRC210) all the samples were previously screened for *BRCA1/2* deleterious variants using SSCP/Heteroduplex analyses and multiplex ligation-dependent probe amplification (MLPA), and were negative for pathogenic or likely pathogenic variants.

### Ethics approval

This study was approved by the Research Ethics Committee of the Faculty of Health Sciences, University of Pretoria (Protocol no. 260/2018). All experiments were performed in accordance with guidelines and regulations. The patients gave written informed consent for participation in the study.

### Analysed cancer genes

The Illumina TruSight Cancer sequencing panel, which targets 94 cancer related genes was used (Supplementary Table S2). All 94 genes were assessed for nonsense, frameshift, or splice-site variants affecting the invariant splice sites. A subset of nineteen established and candidate breast or ovarian cancer genes (*ATM, BARD1*, *BRCA1*, *BRCA2*, *BRIP1, CDH1*, *CHEK2, MLH1, MSH2, MSH6, NBN*, *NF1, PALB2*, *PMS2, PTEN*, *RAD51C*, *RAD51D*, *STK11* and *TP53*) were further investigated for all sequence variants. The results from the previous *BRCA1/BRCA2* screening were also verified.

### Library preparation and sequencing

Patient DNA samples were sent to Omega Biotech in Georgia, USA, where DNA libraries were produced with the TruSight Rapid Capture kit (Illumina) and sequenced using the Illumina TruSight Cancer sequencing panel.

### Sequencing data analysis

All variant calling and variant filtration codes were executed on a Linux cluster with 10× nodes, each having 28× cores and 128 GB of RAM, running CentoS 7.4. Quality analysis of raw sequences was performed using FastQC (version 0.11.7)^58^. Reads were subsequently pre-processed with the FastX toolkit (version 0.0.14) to trim five nucleotides from the 5′- and 3′-ends of the 100 bp paired-end reads^59^. Thereafter, samples were analysed using the GATK best practices^60^ approach by means of the BCBIO pipeline (June 2021 release, detailed tool versions provided in supplementary information)^61^. This includes mapping against the UCSC hg19 reference genome with Burrows–Wheeler Aligner-MEM (BWA MEM)^62^, marking duplicates with Picard and base quality score recalibration. Variant calling was carried out using the HaplotypeCaller in gVCF mode and specified cut off-based filtering of variants done with VariantFiltration using the BCBIO default filtering cut-offs.

#### Variant annotation

Functional variant annotation was done using Variant Effect Predictor (VEP), the default parameters were used in concordance with documentation^63^. Quality-filtered variants were uploaded to the VEP web interface, and additional output fields were activated in the dbNSFP section for LRT_pred^64^, MutationTaster^65^, PROVEAN^66^, CADD^67^ and FATHMM^68^. Filtering of common variants was not performed in VEP.

#### Variant filtration and in silico evaluation of variants

In-house Python code (available on request) was developed for the selection of variants for inclusion in this study. Variants with a minor allele frequency (MAF) of ≥ 1% in the 1000 Genomes African database were removed. For missense variants in the breast cancer susceptibility genes, the results of five in silico functional effect predictors were considered, being LRT_pred^64^, MutationTaster^65^, PROVEAN^66^, CADD^67^ and FATHMM^68^ with variants being selected if at least 3/5 methods predicted a variant to be deleterious. A threshold of 2.0 for GERP_RS and 10.0 for CADD was used. For the other methods, a prediction of ‘D’ was selected. As VEP provides results for multiple transcripts per gene, canonical transcripts are reported on, as determined by mapping of REFSEQ identifiers to Ensembl canonical transcripts via UCSC tables^69^, accessed July 2018.

### Variant classification

Variants were classified in accordance with the American College of Medical Genetics and Genomics guidelines^70^, as pathogenic, likely pathogenic, likely benign, benign or as variants of uncertain significance (VUS). For clarification, pathogenic/likely pathogenic variants were defined as “deleterious variants” linked to the condition “hereditary cancer-predisposition syndrome”.

### Nomenclature

Variants were described according to the Human Genome Variation Society (HGVS) recommendations^71^. Reference sequences were obtained from the NCBI database as listed in Tables 1 and 2. For *BRCA1* the most common human transcript (NM_007294.3) was used with custom numbering of the exons (missing exon 4).

## Supplementary Information

Supplementary Legends.

Supplementary Figure S1.

Supplementary Figure S2.

Supplementary Information.

Supplementary Table S1.

Supplementary Table S2.

Supplementary Table S3.

Supplementary Table S4.

## Data Availability

The raw datasets analysed during the study, and filtering scripts are available from the corresponding author on reasonable request.
