# Supplementary material for: Germline sequence variants contributing to cancer susceptibility in South African breast cancer patients of African ancestry
Source: Sci Rep. 2022 Jan 17;12:802. doi: 10.1038/s41598-022-04791-1 (PMC8763903; doi:10.1038/s41598-022-04791-1)
Supplement: Supplementary file 1 — Supplementary Legends. [file 41598_2022_4791_MOESM1_ESM.docx]

**Supplementary figure and table legends**

Supplementary Table S1. Demographic and clinicopathologic characteristics of the study cohort. * Inf duct = infiltrating ductal carcinoma; inf lobular = infiltrating lobular carcinoma; nos = carcinoma not otherwise specified.

Supplementary Table S2. Alphabetic list of genes analysed with the TruSight cancer panel.

Supplementary Table S3. Benign/Likely benign variants detected.

Supplementary Table S4. Variants in breast cancer susceptibility genes and genes exclusively investigated for truncating variants detected in SA breast cancer cohort of African ancestry.

Supplementary Figure S1: A Venn diagram, indicating the concordance of variant effect predictors predicting a deleterious effect for the main variants of interested presented in the article.

Supplementary Figure S2: A bar graph, indicating the number of variants predicted to be deleterious by each of the variant effect predictors for the main variants of interested presented in the article.
