# Supplementary material for: Germline sequence variants contributing to cancer susceptibility in South African breast cancer patients of African ancestry
Source: Sci Rep. 2022 Jan 17;12:802. doi: 10.1038/s41598-022-04791-1 (PMC8763903; doi:10.1038/s41598-022-04791-1)
Supplement: Supplementary file 2 — Supplementary Figure S1. [file 41598_2022_4791_MOESM2_ESM.docx]

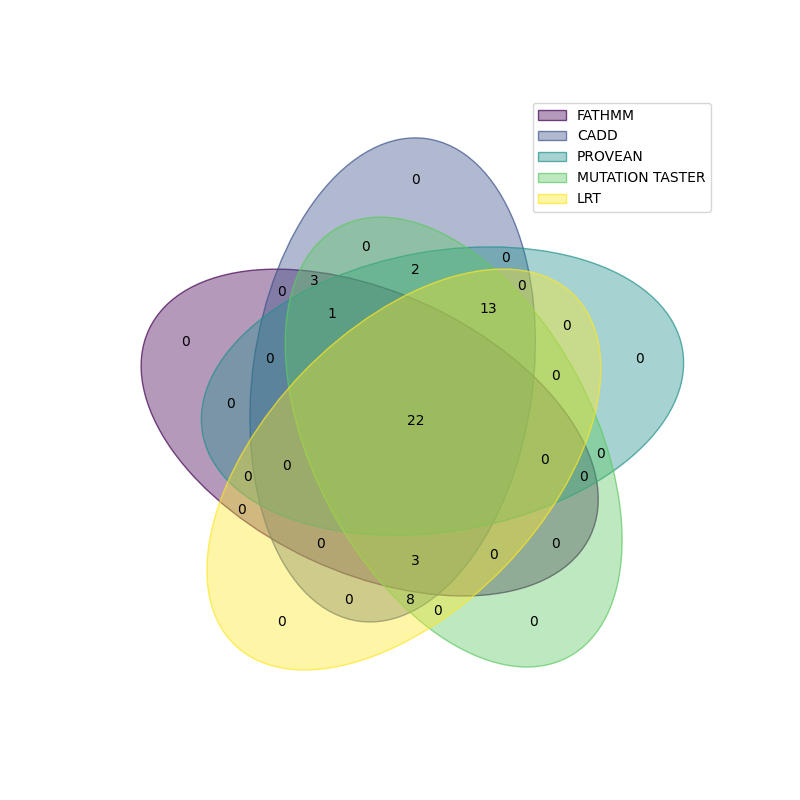


Supplementary Figure S1: A Venn diagram, indicating the concordance of variant effect predictors predicting a deleterious effect for the main variants of interested presented in the article. (Figure generated using Matplotlib 3.4.2: https://matplotlib.org).
