# Supplementary material for: Germline sequence variants contributing to cancer susceptibility in South African breast cancer patients of African ancestry
Source: Sci Rep. 2022 Jan 17;12:802. doi: 10.1038/s41598-022-04791-1 (PMC8763903; doi:10.1038/s41598-022-04791-1)
Supplement: Supplementary file 3 — Supplementary Figure S2. [file 41598_2022_4791_MOESM3_ESM.docx]

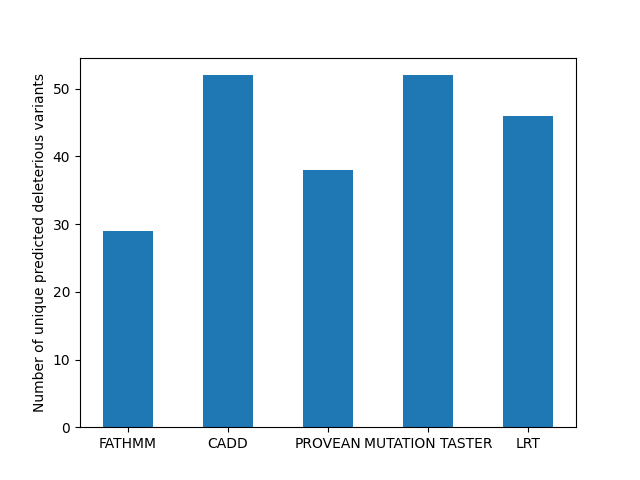


Supplementary Figure 2: A bar graph, indicating the number of variants predicted to be deleterious by each of the variant effect predictors for the main variants of interested presented in the article. (Figure generated using Matplotlib 3.4.2: https://matplotlib.org).
