# Supplementary material for: Germline sequence variants contributing to cancer susceptibility in South African breast cancer patients of African ancestry
Source: Sci Rep. 2022 Jan 17;12:802. doi: 10.1038/s41598-022-04791-1 (PMC8763903; doi:10.1038/s41598-022-04791-1)
Supplement: Supplementary file 4 — Supplementary Information. [file 41598_2022_4791_MOESM4_ESM.docx]

**Supplementary methods**

**VEP annotation**:

GRCh37 26 October 2018

**BCBIO software versions:**

bamtools,2.4.0

bcbio-nextgen,1.0.9a0

bcbio-variation,0.2.6

bcftools,1.6

bedtools,2.27.1

biobambam,2.0.87

bioconductor-bubbletree,2.8.0

bowtie2,2.2.8

break-point-inspector,1.5

bwa,0.7.17

chanjo,

cnvkit,0.9.3

cufflinks,2.2.1

cutadapt,1.16

fastqc,0.11.7

featurecounts,1.4.4

freebayes,1.1.0.46

gatk4,4.0.2.1

gemini,0.20.1

grabix,0.1.8

hisat2,2.1.0

htseq,0.9.1

lumpy-sv,0.2.13

manta,1.3.2

metasv,0.4.0

mirdeep2,2.0.0.7

mutect,1.1.5

novoalign,3.07.00

novosort,V3.00.02

oncofuse,1.1.1

phylowgs,20150714

picard,2.17.11

platypus-variant,0.8.1.1

preseq,2.0.2

qualimap,2.2.2a

rna-star,

rtg-tools,3.8.4

sailfish,0.10.1

salmon,0.9.1

sambamba,0.6.6

samblaster,0.1.24

samtools,1.7

scalpel,0.5.3

seqbuster,3.1

snpeff,4.3i

vardict,2017.11.23

vardict-java,1.5.1

variant-effect-predictor,

varscan,2.4.3

vcflib,1.0.0_rc1

vt,2015.11.10

wham,1.7.0.311

**BCBIO data versions:**

genome,resource,version

hg19,seq,broad-20120813

hg19,twobit,broad-20120813

hg19,GA4GH_problem_regions,20160916

hg19,capture_regions,20161202

hg19,MIG,20150730

hg19,prioritize,20160215

hg19,dbsnp,150-20170710

hg19,hapmap,3.3

hg19,1000g_omni_snps,2.5

hg19,ACMG56_genes,20160629

hg19,1000g_snps,2.8

hg19,mills_indels,2.8

hg19,clinvar,20170905

hg19,cosmic,68-20180114

hg19,ancestral,20141010

hg19,qsignature,20140703

hg19,genesplicer,2004.04.03

hg19,effects_transcript,2017-03-16

hg19,vcfanno,20171008

hg19,viral,2017.02.04

hg19,transcripts,2014-07-17

hg19,RADAR,v2-20180202

hg19,srnaseq,20180122

hg19,giab-NA12878,v3_3_2

hg19,platinum-genome-NA12878,v8_0_1

hg19,giab-NA24385,v3_3_2-sv_v0.5.0

hg19,giab-NA24631,v3_3_2
