# Supplementary material for: Germline sequence variants contributing to cancer susceptibility in South African breast cancer patients of African ancestry
Source: Sci Rep. 2022 Jan 17;12:802. doi: 10.1038/s41598-022-04791-1 (PMC8763903; doi:10.1038/s41598-022-04791-1)
Supplement: Supplementary file 5 — Supplementary Table S1. [file 41598_2022_4791_MOESM5_ESM.docx]

| **BRB lab #** | **Age @ Dx (yrs,months)** | **Ethnicity/**  **Language** | **Family History** | **Histology*** | **Grade** |
| --- | --- | --- | --- | --- | --- |
| **2** | 35, 6 months | Sepedi | None | inf duct | IV |
| **3** | 48 & 10 months | Tswana | None | inf duct | IV |
| **5** | 49, 10 months | N. Sotho | Mother breast ca | inf duct | IV |
| **6** | 43 & 8 months | N. Sotho | None | inf duct | III a |
| **8** | 28, 0 months | N. Sotho | None | inf duct | IV |
| **9** | 36 & 5 months | N. Sotho | None | inf duct | IV |
| **10** | 42, 8 months | N. Sotho | None | medullary ductal | III |
| **11** | 44, 0 months | Tswana | None | inf duct | III |
| **14** | 47 & 8 months | Zulu | None | Unknown | IV |
| **17** | 44 & 11 months | Ndebele | Cousin breast ca | inf duct | II b |
| **18** | 31 & 9 months | Tswana | None | inf duct | III |
| **19** | 36 | Swazi | None | inf duct | IV |
| **20** | 34, 1 months | N. Sotho | None | inf duct | IV |
| **21** | 31 | Unknown | None | medullary ductal | III b |
| **28** | 29, 4 months | Zulu | None | inf duct | III a |
| **34** | 43, 1 month | Tswana | None | inf duct | II |
| **37** | 37, 5 months | Ndebele | Mother breast ca | inf duct | IV |
| **38** | 46, 10 months | Tswana | None | Unknown | II a |
| **39** | 54 | Zulu | None | inf duct | IV |
| **42** | 49, 11 months | Tswana | None | Unknown | II a |
| **44** | 30 | N. Sotho | None | Unknown | ? |
| **46** | 46, 6 months | Tswana | None | inf duct | IV |
| **47** | 52, 8 months | Zulu | None | carcinoma nos | IV |
| **48** | 42, 3 months | Tswana | None | inf duct | II b |
| **49** | 43, 4 months | Zulu | Aunt breast ca | inf duct | II b |
| **50** | 40, 4 months | Tswana | Mother breast ca | inf duct | IV |
| **51** | 52, 6 months | Unknown | None | inf duct | IV |
| **52** | 46, 11 months | Unknown | None | inf duct | II b |
| **53** | 43, 5 months | Zulu | None | inf duct | IV |
| **55** | 32, 9 months | N. Sotho | None | Unknown | II a |
| **57** | 26, 1 month | S. Sotho | None | inf duct | III b |
| **58** | 33, 7 months | N. Sotho | None | inf duct | II a |
| **59** | 39, 0 months | Swazi | None | inf duct | IV |
| **62** | 43, 7 months | N. Sotho | None | inf duct, with tubular differentiation | IV |
| **68** | 43, 0 months | Ndebele | Mother breast ca | inf duct | III b |
| **70** | 42, 7 months | Tswana | Maternal aunt breast ca | inf duct | IV |
| **72** | 53, 8 months | Unknown | None | inf duct | IV |
| **73** | 29, 11 months | Tswana | None | inf duct | II b |
| **74** | 47, 4 months | N. Sotho | None | inf duct | III b |
| **75** | 46, 11 months | Zulu | None | inf duct | II b |
| **77** | 47, 7 months | Zulu | None | inf duct | IV |
| **78** | 36, 4 months | N. Sotho | None | inf duct | III b |
| **81** | 52, 6 months | Sotho | None | invasive lobular | IV |
| **83** | 53, 0 months | Sepedi | None | Unknown | III b |
| **84** | 43, 7 months | Tswana | None | Unknown | II a |
| **87** | 50 | Swazi | None | inf duct | III |
| **88** | 39, 3 months | N. Sotho | None | inf duct | IV |
| **89** | 48, 8 months | N. Sotho | Uncle prostate ca | inf duct | II a |
| **91** | 48, 0 months | Unknown | None | inf duct | III a |
| **94** | 35, 3 months | Zulu | None | inf duct | III b |
| **96** | 30, 5 months | N. Sotho | None | inf duct | IV |
| **98** | 43, 3 months | Tswana | None | inf duct | II b |
| **99** | 45, 3 months | Zulu | None | inf duct | II b |
| **101** | 50, 8 months | Tswana | None | inf duct | IV |
| **102** | 40, 9 months | Zulu | None | inf duct | IV |
| **104** | 47, 0 months | Zulu | None | inf duct with areas of DCIS | II b |
| **106** | 35, 0 months | Unknown | None | inf duct | IV |
| **108** | 39, 6 months | Zulu | None | inf duct | III b |
| **111** | 42, 7 months | Tswana | None | inf duct + DCIS | IV |
| **113** | 41, 7 months | Tswana | None | inf duct | III b |
| **114** | 47, 1 month | Zulu | None | inf duct | II |
| **118** | 33, 10 months | N. Sotho | Father oesophagus ca? | inf duct | IV |
| **120** | 52, 6 months | Zulu | None | inf duct | IV |
| **121** | 54, 0 months | Zulu | None | Unknown | ? |
| **122** | 43, 10 months | Tswana | None | inf duct | IV |
| **123** | 44, 4 months | Unknown | None | inf duct | II |
| **124** | 25, 9 months | Tswana | None | inf duct | IV |
| **125** | 38, 2 months | Tswana | None | inf duct | IV |
| **129** | 43, 2 months | Shangaan | None | inf duct | IV |
| **130** | 45, 8 months | Tswana | None | inf duct | III b |
| **131** | 45, 3 months | Zulu | None | inf duct | IV |
| **132** | 42, 4 months | Unknown | None | inf duct | IV |
| **137** | 48, 0 months | Sotho | None | inf duct | III b |
| **138** | 48, 6 months | Tswana | None | inf duct | III a |
| **139** | 48, 3 months | Tswana | None | inf lobular | IV |
| **142** | 52, 11 months | Unknown | None | inf duct | III a |
| **143** | 42, 0 months | Ndebele | None | inf duct | IV |
| **146** | 52, 2 months | Tswana | None | inf duct | IV |
| **147** | 52, 10 months | Zulu | Mother breast ca | Unknown | IV |
| **148** | 39, 8 months | Ndebele | None | papillary with DCIS | II a |
| **150** | 45, 6 months | Sotho | None | inf duct | II b |
| **152** | 39, 4 months | Sepedi | None | inf duct | II b |
| **153** | 22, 10 months | Tswana | None | inf duct | IV |
| **154** | 51, 2 months | Tswana | None | inf duct | II |
| **156** | 47, 0 months | Unknown | None | inf duct | II b |
| **158** | 53, 7 months | Ndebele | None | inf duct | IV |
| **160** | 40, 3 months | Xhosa | None | inf duct | II a |
| **161** | 29, 6 months | Sotho | None | Unknown | IV |
| **162** | 42, 10 months | Sepedi | Sister breast ca | Unknown | III |
| **166** | 28, 10 months | N. Sotho | Sister & Aunt breast ca | inf duct | III b |
| **167** | 44, 6 months | Unknown | None | inf duct | IV |
| **169** | 38, 1 month | N. Sotho | None | inf duct | IV |
| **170** | 40, 2 months | Sepedi | None | inf duct | II |
| **171** | 37, 4 months | Tsonga | None | inf duct | III |
| **172** | 31, 3 months | N. Sotho | None | inf duct | III a |
| **173** | 34, 9 months | Tswana | None | inf duct | III a |
| **174** | 37, 8 months | N. Sotho | None | inf duct | III |
| **175** | 37, 9 months | Swazi | None | inf mucinous | IV |
| **177** | 26, 5 months | N. Sotho | None | inf duct | II b |
| **182** | 41, 2 months | Tswana | None | inf duct | III |
| **185** | 30, 4 months | Unknown | None | inf duct | IV |
| **186** | 35, 9 months | Sotho | None | inf duct | II b |
| **187** | 41, 2 months | S. Sotho | None | inf duct | IV R |
| **188** | 43, 10 months | Zulu | None | inf duct | II a |
| **189** | 40, 3 months | Tsonga | None | inf duct | III |
| **190** | 38, 9 months | Unknown | None | inf duct | IV |
| **191** | 28, 8 months | Tswana | None | inf duct | II b |
| **193** | 43, 5 months | Ndebele | None | inf duct | II b |
| **194** | 44, 1 month | Sotho | None | inf duct | II b |
| **197** | 51 | Zulu | None | inf duct | IV |
| **199** | 45, 2 months | Zulu | None | inf duct | III b |
| **200** | 41, 6 months | Sepedi | None | inf duct | III a |
| **201** | 42, 4 months | Ndebele | Mother's Aunt breast ca | inf duct | III b |
| **203** | 39, 7 months | Zulu | None | inf duct | IV |
| **205** | 43, 6 months | Tswana | None | medullary ductal | III b |
| **207** | 49, 9 months | Tswana | None | inf duct | IV |
| **208** | 44, 8 months | Zulu | None | inf duct | III a |
| **215** | 40, 5 months | Ndebele | None | inf duct | IV |
| **220** | 38, 11 months | Sotho | None | inf duct | II |
| **224** | 26, 7 months | Swazi | None | inf duct | II a |
| **225** | 34, 4 months | Zulu | None | inf duct | IV |
| **226** | 26, 2 months | Zulu | None | inf duct | III b |
| **229** | 38, 11 months | Zulu | None | inf duct | III |
| **233** | 41, 1 month | Ndebele | None | inf duct | III |
| **234** | 39, 10 months | Tswana | None | inf duct | III a |
| **236** | 47, 8 months | Swazi | None | inf duct | III |
| **237** | 28, 4 months | Zulu | None | inf duct | IV |
| **238** | 44, 10 months | Zulu | None | inf duct | III a |
| **239** | 51, 11 months | Zulu | None | inf duct | III |
| **240** | 48, 10 months | Sotho | None | inf duct | IV |
| **241** | 40, 1 month | Xhosa | None | Unknown | IV |
| **242** | 46, 10 months | Zulu | None | inf duct | II b |
| **245** | 35, 10 months | N. Sotho | Two sisters breast ca | medullary ductal | ? |
| **246** | 42, 1 month | Tswana | Mother breast ca | inf duct | I |
| **249** | 32, 4 months | N. Sotho | None | inf duct | II b |
| **252** | 40, 9 months | Sepedi | None | inf duct | III b |
| **253** | 46, 4 months | N. Sotho | Father stomach ca | inf duct | IV |
| **254** | 49, 0 months | Ndebele | None | inf duct | IV |
| **255** | 51, 4 months | Sotho | None | inf duct | IV |
| **257** | 46, 9 months | N. Sotho | None | inf duct | IV |
| **258** | 28, 1 month | Ndebele | None | inf duct | II a |
| **259** | 26, 11 months | Sepedi | None | inf duct | IV |
| **260** | 39, 0 months | Tswana | None | Unknown | IV |
| **261** | 38, 1 month | Xhosa | None | Unknown | II a |
| **264** | 42, 3 months | Shangaan | None | inf duct | IV |
| **265** | 43, 7 months | Zulu | None | Unknown | IV |
| **267** | 45, 11 months | Tswana - Venda | None | Unknown | II b |
| **268** | 46, 2 months | N. Sotho | None | inf duct | IV |
| **270** | 41, 2 months | Tswana | None | inf duct | IV |
| **271** | 41, 3 months | Zulu | None | inf duct | III |
| **272** | 40, 2 months | Zulu | None | inf duct | II b |
| **273** | 48, 9 months | N. Sotho | Daughter breast ca | inf duct | ? |
| **275** | 47, 4 months | S. Sotho | None | inf duct | IV |
| **276** | 46, 6 months | N. Sotho | None | inf duct | ? |
| **279** | 42, 4 months | Zulu | None | Unknown | ? |
| **281** | 51, 0 months | Zulu | Sister Ovarian ca | inf duct | IV |
| **282** | 49, 11 months | Swazi | None | inf duct | III b |
| **283** | 47 | Tswana | Mother breast ca | inf duct | IV |
| **284** | 30, 10 months | Zulu | None | inf duct | II b |
| **286** | 48, 11 months | Unknown | None | inf duct | III b |
| **287** | 50, 7 months | Ndebele | None | inf duct | III a |
| **288** | 52, 11 months | Swazi | None | inf duct | IV |
| **290** | 26, 4 months | N. Sotho | None | Unknown | III b |
|  |  |  |  |  |  |
| **BRC134** | 38 | Sotho | Mother & sister breast ca | Unknown | ? |
| **BRC210** | 36 | Sotho | Mother & 2 aunts breast ca | Unknown | ? |

**Supplementary Table S1.** Demographic and clinicopathologic characteristics of the study cohort.

* Inf duct = infiltrating ductal carcinoma; inf lobular = infiltrating lobular carcinoma; nos = carcinoma not otherwise specified.
