# Supplementary material for: Germline sequence variants contributing to cancer susceptibility in South African breast cancer patients of African ancestry
Source: Sci Rep. 2022 Jan 17;12:802. doi: 10.1038/s41598-022-04791-1 (PMC8763903; doi:10.1038/s41598-022-04791-1)
Supplement: Supplementary file 6 — Supplementary Table S2. [file 41598_2022_4791_MOESM6_ESM.docx]

*AIP, ALK, APC, ATM, BAP1, BLM, BMPR1A, BRCA1, BRCA2, BRIP1, BUB1B, CDC73, CDH1, CDK4, CDKN1C, CDKN2A, CEBPA, CEP57, CHEK2, CYLD, DDB2, DICER1, DIS3L2, EGFR, EPCAM, ERCC2, ERCC3, ERCC4, ERCC5, EXT1, EXT2, EZH2, FANCA, FANCB, FANCC, FANCD2, FANCE, FANCF, FANCG, FANCI, FANCL, FANCM, FH, FLCN, GATA2, GPC3, HNF1A, HRAS, KIT, MAX, MEN1, MET, MLH1, MSH2, MSH6, MUTYH, NBN, NF1, NF2, NSD1, PALB2, PHOX2B, PMS1, PMS2, PRF1, PRKAR1A, PTCH1, PTEN, RAD51C, RAD51D, RB1, RECQL4, RET, RHBDF2, RUNX1, SBDS, SDHAF2, SDHB, SDHC, SDHD, SLX4, SMAD4, SMARCB1, STK11, SUFU, TMEM127, TP53, TSC1, TSC2, VHL, WRN, WT1, XPA,* and *XPC*

**Supplementary Table S2.** Alphabetic list of genes analysed with the TruSight cancer panel.
