# Supplementary material for: Germline sequence variants contributing to cancer susceptibility in South African breast cancer patients of African ancestry
Source: Sci Rep. 2022 Jan 17;12:802. doi: 10.1038/s41598-022-04791-1 (PMC8763903; doi:10.1038/s41598-022-04791-1)
Supplement: Supplementary file 7 — Supplementary Table S3. [file 41598_2022_4791_MOESM7_ESM.docx]

| **Gene (RefSeq)** | **Variant** | **Predicted protein change** | **dbSNP** | **Patient** |
| --- | --- | --- | --- | --- |
| *ATM* (NM_000051.3) | c.334G>A | NP_000042.3:p.Ala112Thr | rs146382972 | BRB171 |
|  |  |  |  | BRB68 |
| *ATM* | c.2096A>G | NP_000042.3:p.Glu699Gly | rs147934285 | BRB142 |
|  |  |  |  | BRB190 |
|  |  |  |  | BRB91 |
| *ATM* | c.7313C>T | NP_000042.3:p.Thr2438Ile | rs147604227 | BRB171 |
| *BRCA1* (NM_007294.3) | c.4682C>T | NP_009225.1:p.Thr1561Ile | rs56158747 | BRB143 |
|  |  |  |  | BRB146 |
|  |  |  |  | BRB28 |
|  |  |  |  | BRB42 |
| *BRCA2* (NM_000059.3) | c.3858_3860del | NP_000050.2:p.Lys1286del | rs80359406 | BRB59 |
|  |  |  |  | BRB9 |
| *BRCA2* | c.9875C>T | NP_000050.2:p.Pro3292Leu | rs56121817 | BRB160 |
|  |  |  |  | BRB99 |
| *CHEK2* (NM_001005735.1) | c.254C>T | NP_001005735.1:p.Pro85Leu | rs17883862 | BRB172 |
|  |  |  |  | BRB224 |
|  |  |  |  | BRB52 |
|  |  |  |  | BRB62 |
|  |  |  |  | BRB88 |
|  |  |  |  | BRC134 |
| *MSH6* (NM_000179.2) | c.3911G>A | NP_000170.1:p.Arg1304Lys | rs34625968 | BRB87 |
| *NF1* (NM_001042492.2) | c.3169G>A | NP_001035957.1:p.Ala1057Thr | rs1367746167 | BRB108 |
| *NF1* | c.7539G>C | NP_001035957.1:p.Gln2513His | rs2070170345 | BRB108 |
| *PMS2* (NM_000535.6) | c.1268C>T | NP_000526.2:p.Ala423Val | rs756883400 | BRB19 |
| *PMS2* | c.612T>A | NP_000526.2:p.Asn204Lys | - | BRC210 |
| *PMS2* | c.497T>C | NP_000526.2:p.Leu166Pro | rs116349687 | BRB114 |
|  |  |  |  | BRB142 |
|  |  |  |  | BRB225 |
|  |  |  |  | BRB264 |
|  |  |  |  | BRB265 |
| *RAD51D* (NM_002878.3) | c.146C>T | NP_002869.3:p.Ala49Val | rs140317560 | BRB264 |

**Supplementary Table S3.** Benign/Likely benign variants detected.
